# A Rare Report of Lung Metastasis of the Common Non-Melanotic Skin Cancer

**Published:** 2018-01

**Authors:** Mohsen Shafiepour, Arda Kiani, Kimia Taghavi, Sharareh Seifi, Mitra Sadat Rezaie, Seyed Mohammad Reza Hashemian, Atefeh Abedini

**Affiliations:** 1 Chronic Respiratory Diseases Research Center, National Research Institute of Tuberculosis and Lung Disease (NRITLD), Shahid Beheshti University of Medical Sciences, Tehran, Iran,; 2 Tracheal Diseases Research Center, NRITLD, Shahid Beheshti University of Medical Sciences, Tehran, Iran,; 3 Virology Research Center, NRITLD, Shahid Beheshti University of Medical Sciences, Tehran, Iran,; 4 Clinical Tuberculosis center, NRITLD, Shahid Beheshti University of Medical Sciences, Tehran, Iran

**Keywords:** Basal cell carcinoma, Pulmonary, Lung metastasis, Metastatic basal cell carcinoma

## Abstract

Basal cell carcinoma is a common non-melanotic skin cancer with a prevalence of 74.5%–82.6% in the Iranian population. BCC rarely metastasizes. However, metastasis can cause significant morbidity. The prevalence of metastatic basal cell carcinoma varies between 0.0028% and 0.55% of all cases. We describe a case of lung metastasis of basal cell carcinoma of the scalp.

## INTRODUCTION

Basal cells are small, round cells found in the lower layer of the epidermis (1, 2). Basal cell carcinoma (BCC) is the most common non-melanotic skin malignancy and also the most common type of skin cancer (3, 4). BCC is usually characterized by invasive local growth and tissue destruction, with a worldwide prevalence of up to 80% and 74.5%–82.6% in Iran (1, 2, 5, 6). BCC is more common among fair-skinned males and occurs nearly 85% of the time in the head and neck region (7, 8). Similar to squamous cell carcinoma (SCC) and Merkel cell carcinoma (MCC), BCC is distinguished by large, facial, invasive, destructive, ulcerated, long-term, and treatment-resistant scars or lesions (1, 9). The metastatic potential of BCC is 0.0028% (28 cases per 1,000,000 BCC cases)–0.55% (1, 7). Therefore, only a few cases of metastatic BCC (MBCC) have been reported in Iran, none of which demonstrated metastasis to the lung (1, 2).

The current report is from Masih Daneshvari hospital, a referral center for pulmonary and lung diseases in Iran, which describes a rare case of metastasis of BCC to the lung.

## CASE SUMMARIES

On October 2011, a 65-year-old fair-skinned female suffering from a waxy flat scalp lesion presented to Loghman Hakim hospital. There was no history of previous skin cancers. A punch lesion biopsy of 4-mm height and 5-mm depth was performed. Histopathology reported the biopsy specimen as a focal epidermal fibrin leukocyte ulcer. The microscopic examination showed BCC characterized by strands of neoplasm containing round vesiculated hyperchromatic nuclei, clear cytoplasm producing shrunken nests, and sclerotic collagen bundles surrounding the neoplasm strands (10). The patient presented for excision surgery 9 weeks after the initial biopsy. The lesion, with clinically 7-mm marked margins, and full-thickness skin was excised. On pathological examination of the 22- × 12-mm specimen, the tumor was diagnosed as multifocal infiltrative BCC. The patient was administered 500 mg cephalexin and 325 mg acetaminophen to prevent local infection. The liver function tests and other routine blood investigations were within normal limits. Local recurrence appeared at the same site 5, 12, and 18 months after the excision surgery. Several punch lesion biopsies were conducted. Histopathology descriptions reported ulcerated surface tumors; focally invading hypodermis; infiltrative mixed nodular, solid morphemic, and pigmented type of BCC. Moreover, the reports noted that the tumors had focally invaded the hypodermis as M80106: metastatic carcinoma; C44.4: malignant neoplasm of skin of scalp and neck; and M8090/3: basal cell carcinoma (11). The lymphatic vascular invasion was also diagnosed in the last skin biopsy. A second excision surgery was done in 2014, and no evidence of tumor recurrence was detected until 2015. However, on computed tomography, no pulmonary invasion was observed at the time of the last excision.

**Figure 1.**
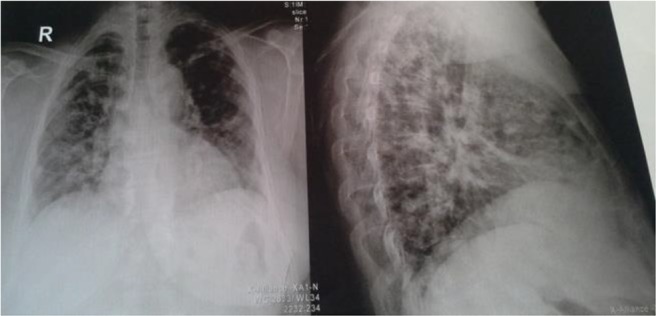
Large lobulated lesions in both the lung lobes in the chest radiography

**Figure 2.**
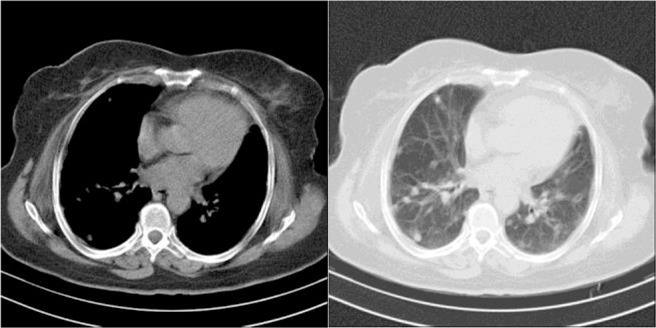
Computed tomogram showing a large solid lobulated margin mass lesion involving the left lung lobe and also the right lung lobe

In September 2015, one year after the second excision surgery, the patient presented to Masih Daneshvari hospital suffering from productive cough and chest pain. No abnormal activity was detected outside the lungs on analyzing the complete blood count and liver function tests. The probability of metastasis was evaluated considering the history of multiple lesions. Accordingly, the patient underwent chest radiography and computed tomography. The chest radiograph demonstrated large lobulated lesions in both lung lobes (Figure 1). Bilateral multiple nodular lung lesions in both the lungs were detected in the computed tomogram (CT scan) (Figure 2). All features suggested a metastatic lesion in the lungs. A CT-guided fine needle aspiration cytology (FNAC) from both lung lesions was performed.

**Figure 3.**
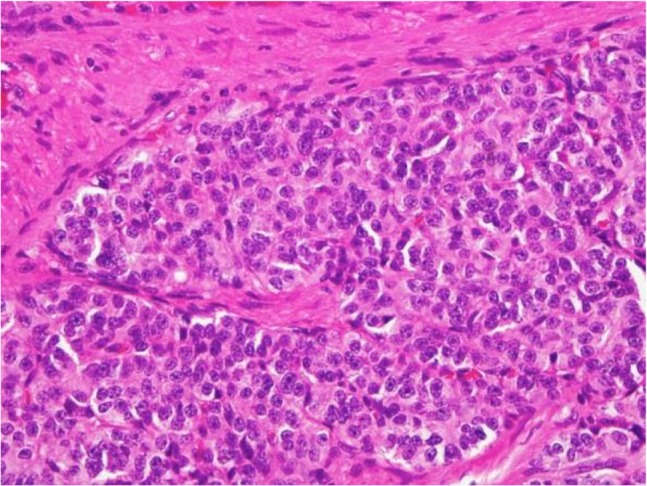
Pathological examination of the CT guided biopsy showed tissue infiltrated by a neoplasm creating nests composed of uniform cells with peripheral palisades which was defined as metastatic basal cell carcinoma (MBCC)

The specimen showed multiple filiform tan-whitish tissues, 4cm in length and 0.1cm in greatest dimension, retained in formalin. The pathological examination of the CT-guided biopsy confirmed that the lung tissue was infiltrated by a neoplasm creating nests composed of uniform cells with peripheral palisades. The CT-guided biopsy and cytology defined positive malignant cells identical with MBCC of the lung (Figure 3).

The specimen was re-examined, and immune histochemical staining was done. The tumor cells were positive for P63 and negative for chromogranin. TTF1, EMA, and TG were negative, which ruled out cutaneous neuroendocrine carcinoma (Merkel cell carcinoma). The findings were similar to the patient’s BCC histological features, which developed five years ago. The similarity of the tumors and histological features indicated that the pulmonary nodules were rare metastatic BCC. Postoperative chemotherapy with 5-fluorouracil (FU) and cisplatin was administered to the patient. In August 2016, after 8 weeks of chemotherapy, on clinical examination, her carcinoma had visibly resolved. The patient’s white blood cell count decreased from 9100 U/L before chemotherapy to 4400 U/L. Also, a follow-up chest CT and radiograph showed an improvement in health. The liver function and routine blood tests showed hyperglycemia and hypertriglyceridemia as side-effects of the chemotherapy. An irregular shaped hyperpigmented macule on the skin of the back of the patient’s hand was diagnosed as a senile lentigo. The results were likely to be age-related.

## DISCUSSION

BCC is most frequently observed in fair-skinned individuals, and 90% occur in sun-exposed areas such as the head and neck region. Three main types of BCC appear to be predominant. The first type manifests as a deep ulcer with raised margins. The second type is a superficial, flat lesion with a waxy surface that is macular and slightly erythematous. The third type appears as a polyploidy tumor with an intact surface. Recurrence and metastasis is generally observed in superficial, infiltrative, micronodular, and morpheaform BCC (1, 2).

MBCC is metastatic, which occurs at distant, non-contiguous sites from the primary cutaneous BCC lesions, with similar histological characteristics to those of the primary BCC (2). MBCC is extremely rare, but it carries high morbidity and mortality rates. Persistence of BCC for many years and recurrence despite optimal treatment are some risk factors predisposing patients to MBCC. Forty-two percent of the MBCC disseminate to the lungs, with symptoms such as productive cough and chest pain (2). In contrast, the current case was reported as a rare pulmonary metastasis due to the relapse of scalp lesion, after five years of repeated diagnosis and rejection.

To our knowledge, no previous case of lung MBCC has been described in Iran.

The therapy of MBCC is surgical excision combined with chemotherapy and radiation therapy. Chemotherapy with cisplatin, either alone or in combination with 5-fluorouracil (FU), cyclophosphamide, cisdiaminedichloroplatinum, vincristine, and bleomycin to overcome resistance has shown significant positive responses (12). Recently, sonidegib, an oral inhibitor, has been approved by the FDA (13). The available data on sonidegib is too limited to determine the overall survival rate. Besides the diagnosis and treatment of MBCC, the prognosis is recommended within five years of cure time.

## CONCLUSION

Although MBCC is a rare entity, its occurrence should be borne in mind, especially when dealing with a giant, recurrent, or long-standing tumor in the head and neck region.
